# A web portal for in-silico action potential predictions

**DOI:** 10.1016/j.vascn.2015.05.002

**Published:** 2015

**Authors:** Geoff Williams, Gary R. Mirams

**Affiliations:** Computational Biology, Dept. of Computer Science, University of Oxford, Oxford OX1 3QD, UK

**Keywords:** High-throughput, Compound screening, Cardiac safety, Open source, Action potential, Mathematical model

## Abstract

**Introduction:**

Multiple cardiac ion channels are prone to block by pharmaceutical compounds, and this can have large implications for cardiac safety. The effect of a compound on individual ion currents can now be measured in automated patch clamp screening assays. In-silico action potential models are proposed as one way of predicting the integrated compound effects on whole-cell electrophysiology, to provide an improved indication of pro-arrhythmic risk.

**Methods:**

We have developed open source software to run cardiac electrophysiology simulations to predict the overall effect of compounds that block I_Kr_, I_CaL_, I_Na_, I_Ks_, I_K1_ and I_to_ to varying degrees, using a choice of mathematical electrophysiology models. To enable safety pharmacology teams to run and evaluate these simulations easily, we have also developed an open source web portal interface to this simulator.

**Results:**

The web portal can be found at https://chaste.cs.ox.ac.uk/ActionPotential. Users can enter details of compound affinities for ion channels in the form of IC_50_ or pIC_50_ values, run simulations, store the results for later retrieval, view summary graphs of the results, and export data to a spreadsheet format.

**Discussion:**

This web portal provides a simple interface to reference versions of mathematical models, and well-tested state-of-the-art equation solvers. It provides safety teams easy access to the emerging technology of cardiac electrophysiology simulations for use in the drug-discovery process.

## 1 Introduction

The heart's pumping action is driven by the flow of electrically charged particles — ions — across the membrane of muscle cells. These ions flow through protein channels in the cell membrane, which change conformation dependent on the voltage (electrical potential due to a difference in charge) across the membrane. The change in conformation makes the ion channels permeable, or not, to the flow of ions between the inside and outside of the cell. If a channel is permeable, then ions passively move through the pore, driven by their concentration gradient and the electrical potential gradient across the membrane. Different ion channels have evolved to be selective to different ionic species (e.g. Na^+^, K^+^, Ca^2 +^, Cl^−^), and to carry these ionic currents with differing time- and voltage-dependence. A number of pumps and exchangers actively move ions back across the membrane to restore intra- and extra-cellular concentrations of ions, enabling sustainable electrical activity.

Ion channels can be blocked by pharmaceutical compounds due to their binding directly to channel pores, or compound binding can lead to conformational changes of the ion channel and also lead to impaired passage of ions. Some cardiac ion channels, such as hERG channels, are particularly prone to block, by a wide variety of pharmaceutical compounds (Mitcheson & Perry, 2003). Blockade of the hERG potassium channel is linked with prolongation of electrical activity at the cell, organ, and body-surface (observed as an increase in the QT interval of the ECG). Both block of hERG and prolongation of QT interval are linked with pro-arrhythmic Torsade-de-Pointes risk (Redfern et al., 2003; Sanguinetti & Tristani-Firouzi, 2006; Pollard et al., 2010). As such, hERG block and human QT intervals are assessed as part of the ICH-S7B and ICH-E14 safety guidelines (ICH, 2005a, 2005b).

Improved predictions of torsadogenic risk have been created using information on a compound's interactions with not simply hERG, but also additional ion channels (Mirams et al., 2011; Kramer et al., 2013). In early drug discovery, compounds are commonly screened for their effect on particular cardiac ion currents using cell lines that over-express certain genes. Table 1 shows common choices for human ventricular targets that routinely feature in pharmaceutical safety screens.

Direct measurements of the overall action of a compound are provided in later safety testing on isolated myocytes, tissue cultures, or ex-vivo cardiac tissue preparations. It would be beneficial to be able to provide these ‘integrated’ predictions for larger numbers of compounds, earlier in drug discovery, prior to such experiments being performed. One way to do this is to let biophysical mathematical models integrate any multi-channel effects of a compound, based on channel screening data.

The targets shown in Table 1 were chosen as potential inputs for simulations because they (i) are important in controlling cardiac electrical activity, (ii) are prone to blockade by pharmaceutical compounds, and (iii) are possible to screen with automated assays.

Additional ion currents can, of course, also be affected by compounds. The late/persistent sodium current *I*_*NaL*_ is of particular interest, as it has been affected by a number of pharmaceutical compounds, and modelling work on block this current has been performed (Noble & Noble, 2006; Moreno et al., 2013). The story is complicated by the fact that late sodium represents just part of the overall sodium current, which may emerge from channel kinetics, or may be carried by voltage-gated sodium channels other than Nav1.5 (Noujaim et al., 2012; Yang et al., 2012). Late sodium does not yet have a standard representation in the mathematical models: sometimes it is a separate current; sometimes late sodium is modelled by preventing inactivation of fast sodium; and sometimes late sodium is represented as a distinct conducting state in a Markov model of the Nav1.5 channel (Irvine, Jafri, & Winslow, 1999). For this reason, introducing late sodium current block into the literature action potential models is not straightforward, and is future work.

The FDA, Cardiac Safety Research Consortium, Health and Environmental Sciences Institute and Safety Pharmacology Society are working on a new Comprehensive in-Vitro Pro-arrhythmia Assay (CiPA). The CiPA initiative intends to use mathematical (in-silico) action potential models to integrate multiple ion channel screening data and to make predictions about pro-arrhythmic risk, to be compared with stem-cell derived myocyte assays (Sager, Gintant, Turner, Pettit, & Stockbridge, 2014). As suggested by some commentaries, the computational models need thorough testing, standardisation and wide availability for such uses (Gintant, 2012; Kleiman, Shah, & Morganroth, 2014; Cavero & Holzgrefe, 2014). To this end, this article introduces a publicly accessible open-source web portal we call ‘AP predict online’. The portal has been developed to enable electrophysiology simulations to be performed by safety teams, to evaluate the performance of different models and to define suitable contexts of use.

## 2 Methods

### 2.1 Mathematical electrophysiology models

Mathematical models of cardiac electrophysiology offer a way to integrate the effect of blocking individual types of cardiac ion current, in order to predict effects at the whole-cell level, and higher. The models are designed to describe the evolution of the cell's electrical activity due to the interaction of the different ionic currents. The electrical activity is most commonly described by the *action potential* — the activation and recovery (known as de- and re-polarisation) of transmembrane voltage.

The models therefore describe the evolution of membrane voltage through time by modelling the membrane as simply a capacitor, and saying “the change in voltage is proportional to the sum of the ionic currents across the membrane”. This is expressed quantitatively as an ordinary differential equation:

(1) $$\frac{dV}{dt}=-\frac{1}{C_{m}}\left(\sum_{\mathrm{channelsj}}I_{j}+I_{\mathrm{stim}}\right)\text{.}$$

Here *V* is the transmembrane voltage, *t* is time, *C*_*m*_ is the capacitance of the membrane, *I*_*j*_ represents each type of ionic current *j*, and *I*_*stim*_ is any stimulus current applied to the cell. This can become a complicated system of nonlinear equations when we consider that the ionic currents *I*_*j*_ are themselves nonlinear functions of both voltage and time.

**Fig. 1:**
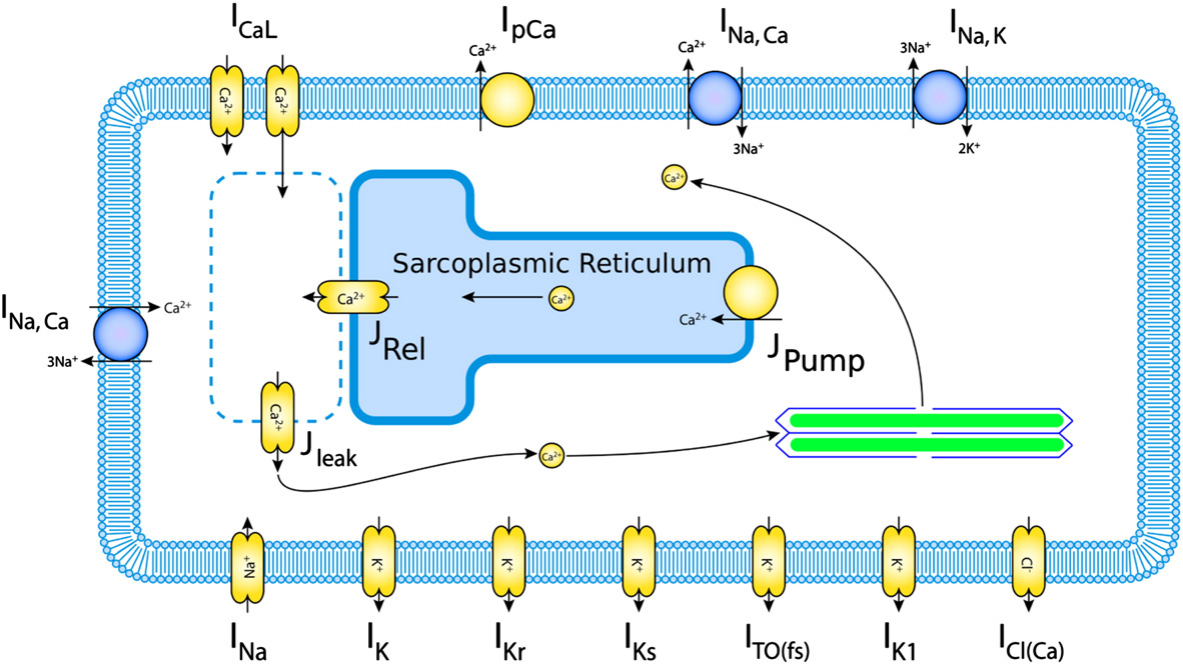
Currents included in the (Shannon et al., 2004) rabbit ventricle model. Ionic pumps are represented by yellow circles, and exchangers by blue circles, the rest of the components in the membrane are ion channels. Currents are given their usual abbreviations, as shown in Table 1. Image adapted from the Physiome Repository http://bit.ly/1J9Z9UI.

This forms a nonlinear system where intuition often fails us, and so quantitative models have allowed much progress, beginning with the Nobel prize winning work of Hodgkin and Huxley (1952), and its application to cardiac cells by Noble (1960). Many of the large advances since — discoveries of new currents, and uncovering of the roles of ionic currents in arrhythmia mechanisms — have been enabled by these mathematical modelling efforts (Noble & Rudy, 1783). Modern mathematical models now include all of the major cardiac ion channels, pumps and exchangers, as well as a detailed description of the calcium subsystem, and the concentration of ions in different cellular compartments. As an example of a modern model, the currents that are modelled in the Shannon, Wang, Puglisi, Weber, and Bers (2004) rabbit ventricle model, available for AP-predict simulations, are shown in Fig. 1.

The AP-predict web portal provides an interface to a simulation tool that attempts to predict changes to the cellular action potential, given the data we have already obtained from cardiac ion channel screens in Table 1. At present the following models are available from the web portal: rabbit — Shannon et al. (2004), Mahajan et al. (2008); human — Ten Tusscher and Panfilov (2006), Grandi, Pasqualini, and Bers (2010), O'Hara, Virág, Varró, and Rudy (2011); and human stem-cell derived myocyte (Paci, Hyttinen, Aalto-Setälä, & Severi, 2013). These models have been chosen to represent the assays that are commonly performed and of safety interest, and to include some of the models that have been used to simulate pharmaceutical compound block in the literature (discussed in Section 3). Any further models that are in the Physiome Model Repository could be added easily to future versions of the portal, and the authors will be pleased to assist with this.

### 2.2 Data for model input

**Fig. 2:**
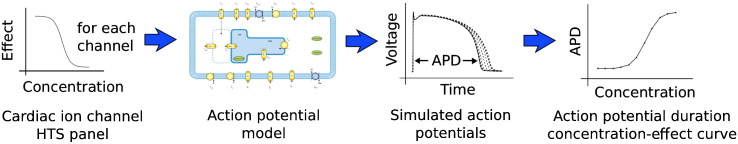
Inputs and outputs of the action potential simulations. Left: ion current concentration–effect curves are taken from each high throughput screen (HTS) and used to calculate a degree of block at any given concentration. The percentage block is applied to each current in a mathematical model ventricular electrophysiology. We then simulate steady pacing (up to a given time limit), and calculate the action potential and examine its duration. The process is repeated for a range of concentrations, and the results are plotted in a summary graph.

AP-predict uses simple concentration–response curves to determine the degree of reduction to be applied to each channel's maximal conductance, for any given concentration. The simulations integrate the concentration–effect curves from multiple ion channel screens (for any channel listed in Table 1), to predict the effect on the whole cell level, as shown in Fig. 2.

A concentration–response (or concentration–effect) curve is commonly defined as:

(2) $$\%\;\mathrm{current}\;\mathrm{remaining}=\frac{100\%}{1+{\left(\frac{\left[\mathrm{Conc}.\right]}{\left[IC_{50}\right]}\right)}^{\mathrm{Hill}}}\text{.}$$

**Fig. 3:**
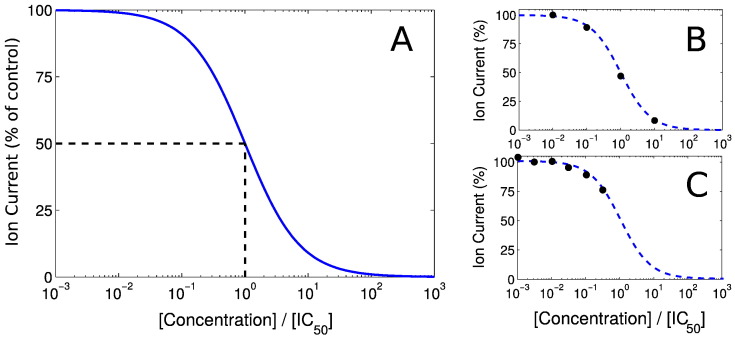
Use of IC50 values to parameterise concentration–effect curves. A: plot of the concentration–response curve given by Eq. (2) with Hill = 1; note that 50% block occurs when [Concentration] ÷ [IC_50_] = 1. B: a curve fitted to data spanning the effect range. C: a curve fitted to data that are all below 50% inhibition.

Eq. (2), plotted in Fig. 3A, provides a very accurate description of (peak) ion-current blockade for most compounds. In the (Elkins et al., 2013) study, we found large variability was associated with Hill coefficient measurements from high-throughput screens. We believe this variability is likely to be a larger source of error than simply saying that “the Hill coefficient is equal to one” in most cases (as from first principles, Hill = 1 occurs when one compound interacts with one ion channel and a channel can be fully blocked by a single molecule of the compound).

Our concentration–response curve is therefore fully defined by a single IC_50_ value — that is, the concentration of the compound that would inhibit the maximum current by 50%. The web portal should be provided with IC_50_ (or pIC_50_) values that result from data fitted to this curve, as shown in Fig. 3B. Where an IC_50_ is not directly observed (e.g. you only know that IC_50_ < 30 μM, since at 30 μM 50% block was not achieved), you should still input the ‘extrapolated’ IC_50_ that parameterises the concentration–response curve fitted through the available data points, as shown in Fig. 3C. If no IC_50_ is provided then the default action is that no block is applied to that channel.

In terms of assay choice, manual whole-cell patch voltage clamp data remains the gold standard and would therefore be the best option (as used in the Mirams et al. (2011) Torsade risk prediction study). Where these data are not available, in earlier drug discovery perhaps, we have also examined using data from high-throughput automated patch clamp screens, as discussed in Section 3.

### 2.3 Assumptions

The following quotation from James Black is a perfect summary of the role of mathematical models in this context.

“[Mathematical] models in analytical pharmacology are not meant to be descriptions, pathetic descriptions, of nature; they are designed to be accurate descriptions of our pathetic thinking about nature. They are meant to expose assumptions, define expectations and help us to devise new tests.”

Black (1989)

So in-silico action potential models should not be thought of as performing a simulation that says “*this will be the result of a later experiment*”. Rather they provide a way to define our expectations: “*this is the effect you would expect to see in a later experiment if the compound blocks cardiac ion channels as detected by the early ion-channel screens, and the electrophysiology of the cell works the way we think it does*”. The act of modelling therefore forces us to be explicit about our assumptions and approximations in the safety testing and modelling processes, which we examine further below.

We have only considered the compound's action on the screened ion channels. Clearly, if the compound affects other channels, the integrated response will not be predicted accurately. In addition, chronic changes to an ion current (due to changes in trafficking or other regulation of ion channel expression and/or function) are not captured in the data providing inputs into the simulations.

Each IC_50_ that is used is assumed to be accurate. But we know that the high-throughput automated platforms in particular have some limitations: many operate at room temperature rather than physiological temperatures; and many compounds can bind to the plastics used in piping and wells, which lowers the effective concentration that is tested. As discussed earlier, there is also significant variability associated with IC_50_ values from many of these screens (Elkins et al., 2013).

We have assumed ‘conductance block’ of ion currents. That is, the compound does not preferentially bind to the ion channel in any particular conformation. Certain compounds break this assumption, when binding has a dependence on channel state and/or membrane voltage. This is one mechanism by which ‘use [pacing rate]-dependent’ block can arise. Additionally, we are assuming that a compound does not affect the ion current's voltage-dependent behaviour (rates of kinetic transitions between ion channel states) once it has bound. The portal only considers antagonistic blockade and reduction of the ion channel currents, agonist action is also possible (but rarer), and could be included in future versions.

Simulations apply the steady-state level of block instantaneously, we do not model the accumulation of the compound in the tissue, or the time-dependence of its binding to ion channels. Note that the simulation still has to be run for a large number of paces, as instant steady-state block of channels will not lead to instant steady-state changes in the resulting action potential model (this slow adaptation of the action potential to interventions is also observed in experiments).

We are predicting the percentage change in single-cell action potential duration, we typically infer that the change in ventricular QT interval on an ECG should be similar. Early tissue simulation work shows that there is an excellent correspondence between the two, but QT effects may be slightly larger than action potential changes on average.^1^

The ion channels that were screened in over-expression systems were human isoforms, not animal proteins that can often be found in later safety tests. So, if anything, on this point we might expect to be able to provide better simulation predictions in the human rather than animal situation.

### 2.4 Technical implementation

The underlying simulation software (‘AP predict’) and the web portal (‘AP-predict online’) are released as open source software under the BSD 3-clause licence.^2^

#### 2.4.1 Simulation software

The action potential prediction software ‘AP predict’ is a bolt-on C^++^ project to the open-source and well-tested ‘Chaste’ computational biology library (Mirams et al., 2013; Pathmanathan & Gray, 2013), both are available to download via https://chaste.cs.ox.ac.uk/trac/wiki/ApPredict.

A brief discussion of the steps involved within AP predict follows. The Physiome Model Repository^3^ contains machine-readable descriptions of each model's equations in CellML format (Lloyd, Lawson, Hunter, & Nielsen, 2008), and ‘AP predict’ is shipped with a copy of some of these CellML files. PyCML is used to convert CellML model representations into Chaste C^++^ files (Cooper, Corrias, Gavaghan, & Noble, 2011). ‘CVODE’ is then used to solve the differential equations (Hindmarsh et al., 2005), with variable-order Backwards Differentiation Formulae solvers, taking adaptive time steps, with settings for absolute and relative tolerances of 10^− 6^ and 10^− 8^ respectively. This solver showed the best performance for cardiac electrophysiology amongst the suite of Chaste differential equation solvers in a recent benchmark study (Cooper, Spiteri, & Mirams, 2014). We simulate multiple paces, until a pseudo-steady response to the requested frequency has emerged (i.e. the same behaviour on subsequent paces). This steady state is defined as when the Euclidean (*L*^2^) norm of the vector of ODE state variable changes between paces is less than 10^− 6^. Output is requested every 0.1 ms for the steady-state pacing action potential waveform (dictating the accuracy of action potential duration calculations), and the output is then down-sampled for fast visual presentation in the web portal.

#### 2.4.2 Web portal

The ‘AP-predict online’ web portal is also free open source software.^4^ The portal has three principal components: the ‘client-direct’ component which stores simulation results and generates HTML for rendering the portal web page in a web browser; the ‘app-manager’ component which invokes the ‘AP predict’ simulation software and monitors simulation progress; and finally the ‘AP predict’ simulation software itself (discussed above). The client-direct and app-manager software are written in the ubiquitous Java language as Java servlets, and make extensive use of the popular Spring Framework.

The client-direct and the app-manager components communicate via web services; the benefit of this is that the two components do not need to be running on the same machine. As each simulation is compute-intensive and may take up to ten minutes to complete, the option to spread the work means that the portal page can remain responsive whilst simulations take place.

### 2.5 Security and privacy

Users can register for free on the public portal running on Oxford servers at https://chaste.cs.ox.ac.uk/ActionPotential, via the ‘Registration Form’ button on the portal homepage. A user name and password for log in will then be sent to the user by e-mail. Once logged in, the portal ensures your simulation inputs and results are accessible to you alone. Whilst we do not intend to analyse the information that is entered, information is necessarily stored in a database on the Oxford server to allow you to view results and revisit past simulations, and this database is accessible to the software development team. The portal runs via ‘https’; the standard secure protocol for preventing any information being intercepted between your web browser and the Oxford servers.

We would still strongly recommend that sensitive proprietary information is not entered into the public portal, in case of the unlikely event that this server is compromised. However, since the portal itself is open source software, one could set up a private copy of the portal inside a firewall on an intranet, which some industrial users may wish to do for increased data security. In future we will be releasing additional template code that, when configured, will allow fire-walled copies of the portal to query internal databases of IC_50_ results, after further trials of this with pharmaceutical partners.

## 3 Results

**Fig. 4:**
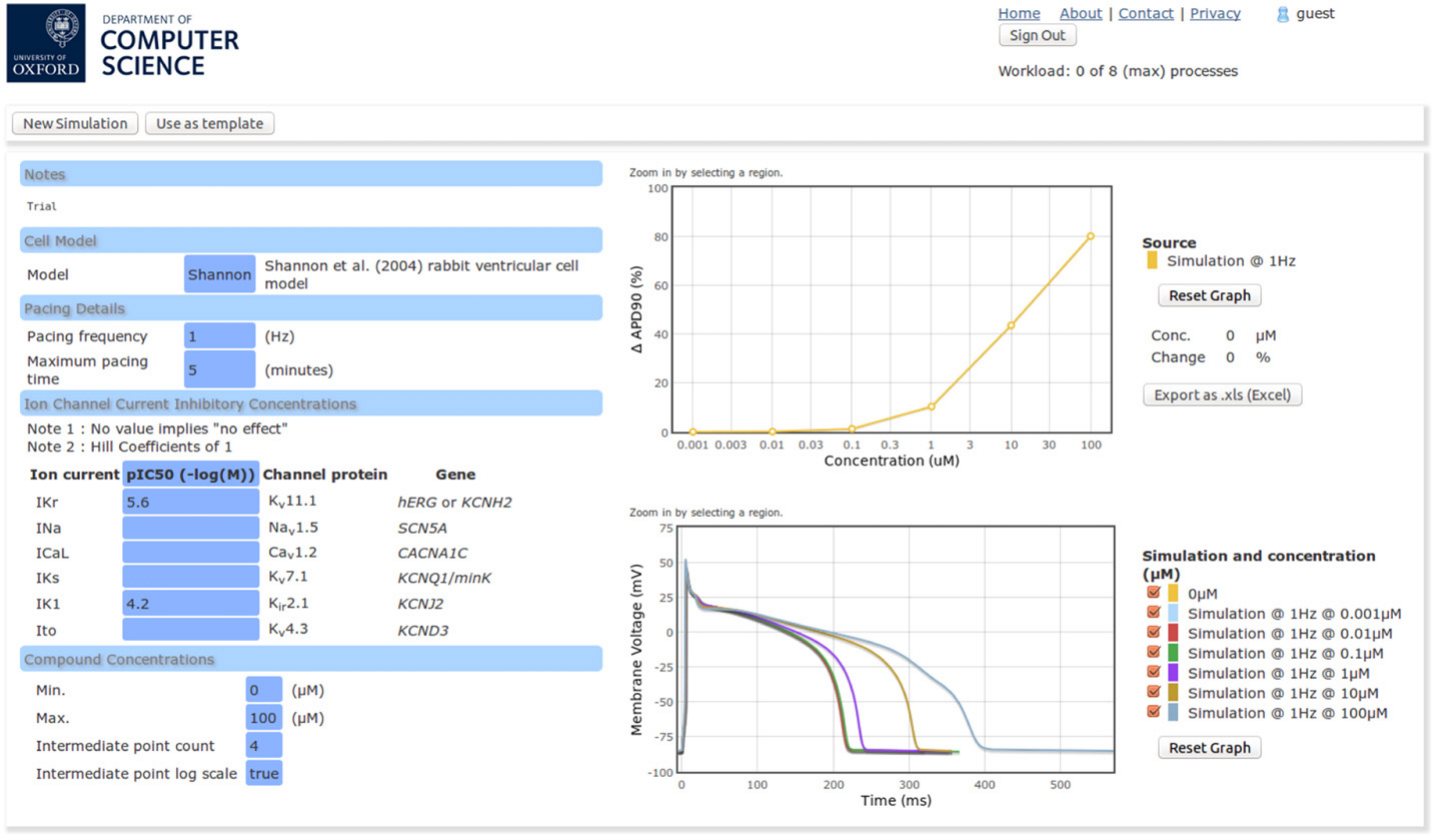
A screen shot of the web portal presentation of simulation results. On the left is a summary of the inputs into the simulations: the model; pacing details; channel pIC _50_ values; and concentration ranges. On the right is a summary plot of percentage changes to APD _90_, and a plot of the steady-state action potential at each test concentration.

The web portal provides a simple interface to simulation results, for each user to retrieve easily at a later date. An example of the web portal results page can be seen in Fig. 4. All of the results that are displayed can be exported in a spreadsheet file format.

Should the results exhibit unusual action potential behaviour such as alternans, or failure to de/re-polarise, then the user's attention is drawn to this via an additional ‘messages’ button next to the results.

A number of evaluations have been performed of the web portal predictions. In Mirams et al. (2011) we showed an excellent correlation between clinical torsadogenic risk and APD_90_ predictions from the Grandi et al. (2010) model, based on (manual) patch-clamp hERG, CaV1.2 and NaV1.5 IC_50_ data, when simulating the compound effect at clinical maximum effective free plasma concentrations.

In a more thorough evaluation of direct portal predictions, we ran predictions of the GlaxoSmithKline rabbit wedge assay for a large number of proprietary compounds (Beattie et al., 2013). We examined whether a compound caused over 10% QT prolongation (or shortening) at any of the concentrations at which it was tested, and then compared whether or not the APD_90_ simulation result, based on screening data, showed the same. Simulations based on medium-throughput PatchXpress screens reached 75% accuracy at predicting this prolongation in the rabbit wedge.

We considered how to measure and account for uncertainty in ion channel screening in Elkins et al. (2013), that study provides a method for determining the subsequent uncertainty in simulation predictions. These simulation confidence intervals will be added to the AP-predict web portal where possible in future versions.

In Mirams et al. (2014) we have compared APD_90_ predictions from three human models (all available in the portal) with the results of human clinical Thorough QT studies. There is a good correspondence, although simulations tended to underestimate the effect that was observed at the estimated clinical free drug concentration. We suspect this could be due to uncertainty in free drug concentration, since similar simulations estimated the concentration at which 10% prolongation occurred much more accurately in the ex-vivo rabbit wedge study discussed above, see Beattie et al. (Fig. 2, 2013).

## 4 Discussion

There are two main contexts of use in which the portal may be helpful: as soon as ion channel screens have been performed; and later when isolated myocyte or tissue-based experiments have been performed. We will discuss these cases separately below.

### 4.1 Before further experiments

The portal can be used to put together the information from a cardiac ion channel screening panel and define expectations of the likely total effect, in different situations. For example predictions can be made for different pacing rates (even though the IC_50_ does not capture use dependence, differences in the contribution of currents at different pacing rates is reflected in the mathematical models). Predictions can also be made for different species or cell types. In the context of the CiPA effort, the means we can make predictions for both stem-cell derived myocytes and adult myocytes. Where it is not feasible to progress all compounds to further experiments, results of the simulations can be used to rank compounds in terms of potential QT liability. The portal predictions are also helpful in designing further experiments by selecting the most likely compound concentrations for observable effects.

### 4.2 After further experiments

At this time, we believe electrophysiology models may be most useful in the context of further experiments, particularly stem-cell derived myocyte assays in the new CiPA paradigm. We discuss the consequences of agreement or disagreement with further experiments below.

#### 4.2.1 Agreement

If the AP-predict prediction is strongly aligned with the further experimental results (in animal tissue or stem-cell derived myocytes for example), this provides strong evidence that we *know the likely APD*/*QT effects*, and we *also know why* they occur. That is, we know how much the compound blocks particular ion channels, and we understand *mechanistically* and *quantitatively* how this leads to APD/QT changes. This provides us with an increased degree of confidence in predicting any clinical effect in human, especially via simulation of the adult human ventricular response.

### 4.2.2 Disagreement

A large disagreement between simulation and experiment is likely to indicate additional compound effects that have not been captured by the ion channel screens. When comparing simulations at the single cell level with tissue experiments it is important to remember that tissue-level effects, such as blockade of gap junctions between cells could also be playing a role, but this should generally be detected via QRS widening or similar measures of conduction velocity. Most disagreements between simulation predictions and experimental results probably mean that one of the assumptions/approximations listed in Section 2.3 has been violated.

An alternative explanation is that the mathematical model that is used is not capturing the interaction of the currents that make up the action potential properly. We cannot yet rule this out, and work is ongoing to improve the model(s). We would suggest that thorough evaluations be performed with each model for each ‘context of use’. For example, this ‘model error’ is thought to be a small effect when predicting rabbit wedge results with the Shannon model, due to the excellent correspondence between simulation and experiment for over 50% of the > 100 compounds we have evaluated to date with GSK (Beattie et al., 2013).

### 4.3 Future work

The web portal is a simplified version of a more fully-featured and database-integrated portal which remains under development. We are in the process of incorporating uncertainty in IC_50_ inputs into the web portal, to provide a confidence interval on predictions. This requires a large number of repeated experiments for one compound on each specific screening assay, the portal will need to include a new calibration step to analyse the spread of such data.

An important task at present is to assess the most predictive in-silico pro-arrhythmic risk markers, other than simply action potential duration. This is an active area of our research. A discussion of possible metrics was given at a recent CiPA update meeting (Mirams, 2015). As new markers of clinical pro-arrhythmic risk become available, we will adapt the portal to perform these simulations and provide an indication of pro-arrhythmic risk alongside the predicted action potentials.

We plan to work on the problem of capturing detailed kinetics of ion channel block in mathematical models of compound action. The mathematical modelling community will need to work with cardiac safety teams to establish the best voltage protocols to measure such details, and to standardise the model representations for automated inclusion in action potential model predictions.

### 4.4 Summary

In summary, there is strong evidence that simulations of the effect of ion channel block on cellular action potential can provide valuable insight on the combined multiple ion-channel action of a novel compound, and subsequent consequences for its cardiac safety. The web portal that we have presented here allows such simulations to be performed freely and easily.

## Figures and Tables

**Table 1:** Assumptions of the cardiac currents that are recorded from cell lines expressing certain genes. Each of these can be given an IC_50_ value in web portal simulations, which is then used to calculate conductance-block in action potential simulations.

Gene	Protein	Current	Current description
hERG or KCNH2	Kv11.1	*I*_*Kr*_	Rapid delayed rectifying potassium current
CACNA1C	Cav1.2	*I*_*CaL*_	L[ong-lasting]-type calcium current
SCN5A	Nav1.5	*I*_*Na*_	[Fast] sodium current
KCNQ1/minK	Kv7.1	*I*_*Ks*_	Slow delayed rectifying potassium current
KCNJ2	Kir2.1	*I*_*K*1_	Inward rectifier potassium current
KCND3	Kv4.3	*I*_*to*,*fast*_	Fast transient outward potassium current

If a model only contains total *I*_*to*_ then this is conductance blocked instead of *I*_*to*,*fast*_.

## Footnotes

1 see an informal blog entry on this: http://mirams.wordpress.com/2014/03/21/apd_vs_qt/ for more information.

2 http://opensource.org/licenses/BSD-3-Clause.

3 https://models.physiomeproject.org/electrophysiology.

4 Available from https://bitbucket.org/gef_work/ap_predict_online.
